# Cost-effectiveness of ferric citrate hydrate in patients with iron deficiency anemia

**DOI:** 10.1007/s12185-024-03905-x

**Published:** 2024-12-26

**Authors:** Mikio Momoeda, Kyoko Ito, Sachie Inoue, Hidetoshi Shibahara, Yuko Mitobe, Norio Komatsu

**Affiliations:** 1 Aiiku Maternal and Child Health Center, Aiiku Hospital, 1-16-10 Shibaura, Minato-Ku, Tokyo, 105-8321 Japan; 2 Medical Affairs Department, Torii Pharmaceutical Co., Ltd., 3-4-1 Nihonbashi-Honcho, Chuo-Ku, Tokyo, 103-8439 Japan; 3 CRECON Medical Assessment Inc., The Pharmaceutical Society of Japan, Nagai Memorial Hall 2-12-15, Shibuya, Shibuya-Ku, Tokyo, 150-0002 Japan; 4 Department of Hematology, Juntendo University Graduate School of Medicine, 2-1-1, Hongo, Bunkyo-Ku, Tokyo, 113-8421 Japan; 5 Department of Advanced Hematology, Juntendo University Graduate School of Medicine, 2-1-1 Hongo, Bunkyo-Ku, Tokyo, 113-8421 Japan

**Keywords:** Oral iron preparation, Ferric citrate hydrate, Iron deficiency anemia, Gastrointestinal adverse events, Cost-effective

## Abstract

We investigated the cost-effectiveness of treating iron deficiency anemia (IDA) with ferric citrate hydrate (FC) in Japan. We employed four treatment strategies: switching from sodium ferrous citrate (SF) to FC at (1) 500 mg (approximately 120 mg of iron) per day or (2) 1000 mg (approximately 240 mg of iron) per day in patients with SF-induced nausea/vomiting, or starting treatment with FC at (3) 500 mg/day or (4) 1000 mg/day. We evaluated the cost-effectiveness of these strategies compared with SF 100 mg (100 mg of iron) per day. Incremental effects over 26 weeks relative to SF 100 mg were 0.0052 quality-adjusted life years (QALYs) for (1) and (2), and 0.0044 QALYs for (3) and (4). From the payer’s perspective, incremental cost-effectiveness ratios (ICERs: JPY/QALY) against SF 100 mg were: (1) 1,107,780, (2) 2,257,477, (3) 5,588,430, and (4) 11,544,816. All four FC strategies were dominant (less costly and more effective) from a limited societal perspective. Treatment with FC for IDA was cost-effective (ICER ≤ JPY 5,000,000/QALY) when switching strategies from the payer perspective, and cost-saving (all FC strategies) from limited societal perspectives. Individual patients' characteristics and cost-effectiveness should be considered in treatment selection.

## Introduction

Iron deficiency anemia (IDA) is the most frequent type of anemia associated with iron deficiency-related decrease in hemoglobin (Hb) production by erythropoiesis [1]. Etiological factors include insufficient dietary iron intake or absorption, greater iron demand during pregnancy, and menstruation-related increase in iron loss [2]. IDA causes symptoms, such as headache, fatigue, and paleness; reduces quality of life (QOL); decreases work productivity; and increases mortality [1, 3–5].

For IDA management, iron preparations are administered in addition to treatment for the primary disease causing IDA and iron intake through dietary counselling. The continuation of treatment for approximately 3 months after the normalization of Hb levels is recommended to ensure adequate iron replacement [6]. Oral iron preparations are generally used as the first-line therapy for IDA; however, gastrointestinal adverse events, such as nausea and vomiting (nausea/vomiting), develop in up to 30% of patients receiving oral iron preparations [7, 8]. In the treatment of IDA requiring continuous medication, the gastrointestinal adverse events of nausea/vomiting have a negative impact on QOL and work productivity [9, 10], which consequently reduces treatment adherence [11, 12]. There is a growing demand for oral iron preparations that cause fewer gastrointestinal adverse events such as nausea/vomiting.

Ferric citrate hydrate (FC, Riona^®^, 250-mg tablet, Torii Pharmaceutical Co. Ltd., Tokyo, Japan) was approved as an oral iron-based phosphate binder for hyperphosphatemia in patients with chronic kidney disease in Japan in 2014. An additional indication for IDA was approved in 2021. A 7-week phase III randomized double-blind study on FC in patients with IDA, using sodium ferrous citrate (SF) at 100 mg/day (containing 100 mg of elemental iron) as a control drug, reported non-inferior changes in Hb levels with FC compared with SF [13]. Moreover, there was a significantly lower incidence of gastrointestinal adverse events, such as nausea/vomiting, observed with FC doses of 500 mg/day (containing approximately 120 mg of elemental iron) and 1000 mg/day (containing approximately 240 mg of elemental iron).

The price of FC per 250-mg tablet in Japan in the fiscal year of 2023 was JPY 74.1 (1USD = JPY 141.56; yearly-average annual TTS (telegraphic transfer selling rate) for 2023 by Mitsubishi UFJ Research and Consulting), whereas that of SF per 50-mg tablet ranged from JPY 5.7 to JPY 7.2 [14]. FC is more expensive than SF, for which generic products are readily available. However, FC is associated with fewer gastrointestinal adverse events, such as nausea/vomiting, leading to an anticipated improvement in adherence. Furthermore, nausea/vomiting as a result of the administration of iron preparations in patients with heavy menstrual bleeding (HMB) or anemia have a negative impact on QOL and work productivity. The rate of presenteeism, defined as the issue of workers being present on the job but not fully functioning due to illness or other medical conditions [15], was significantly higher in the presence of nausea/vomiting compared with their absence [9, 10]. Therefore, a reduction in the incidence of nausea/vomiting associated with FC administration may lead to decreased work productivity losses associated with nausea/vomiting [9, 10].

We evaluated the cost-effectiveness of treating IDA with FC in Japan from payer’s and societal perspectives based on clinical evidence obtained in a phase III study on FC in patients with IDA.

## Materials and methods

### Overview and model structure

The target population for the analyses was adult Japanese IDA patients with a mean age of 40.7 years, taken from a previously reported phase III study on FC [13]. Based on the treatment modalities for IDA with iron preparations, the cost-effectiveness of the following four treatment strategies using FC relative to that of SF 100 mg/day was evaluated: (i) treatment initiated at 100 mg/day of SF and switched to 500 mg/day of FC if nausea/vomiting occurs [SF-FC 500 mg], (ii) treatment initiated at 100 mg/day of SF and switched to 1000 mg/day of FC if nausea/vomiting occurs [SF-FC 1000 mg], (iii) treatment initiated at FC 500 mg/day only [FC 500 mg], and (iv) treatment initiated at FC 1000 mg/day only [FC 1000 mg].

Analyses were conducted in accordance with the Japanese cost-effectiveness analysis guideline [16]. Only direct medical costs were considered from the Japanese public healthcare payer’s perspective. Work productivity loss was considered in addition to medical costs from limited societal perspectives because nausea/vomiting related to the administration of iron preparations affects work productivity [10]. The health outcomes of each intervention were assessed in terms of quality-adjusted life years (QALYs). The duration of the analyses was set for 26 weeks based on the 7-week period used in the phase III study, a 7-week switching period, and 12 weeks after the normalization period of Hb to ensure adequate iron replacement [6]. Since the analysis period was short, a discount rate was not applied.

**Fig. 1:**
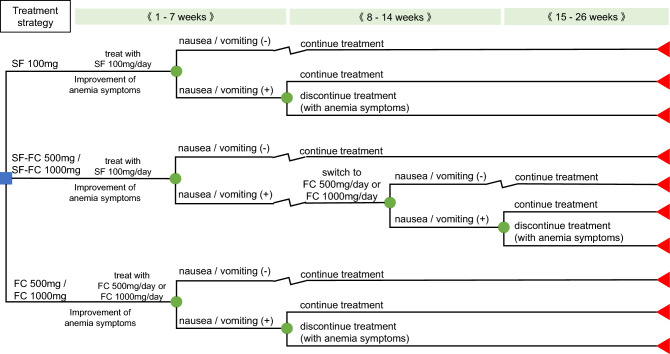
Model structure. FC: ferric citrate hydrate, SF: sodium ferrous citrate

A decision tree model based on the evaluation period of the phase III study on FC [13] was used to evaluate the cost-effectiveness of the four FC treatments relative to SF 100 mg/day (Fig. 1). The analyzed population received treatment with SF or FC at the initiation of the analysis according to the four treatment strategies. The attenuation of anemia or the development of nausea/vomiting while taking SF or FC were considered. In drug-switching strategies (SF-FC 500 mg and SF-FC 1000 mg), patients who developed nausea/vomiting were switched to FC after administration of SF for 7 weeks. The discontinuation of treatment in patients who developed nausea/vomiting was also considered after administration of SF or FC monotherapy for 7 weeks. Nausea/vomiting continues for a specific period unless the causative drug is switched or discontinued. Although nausea/vomiting disappears when treatment is discontinued, the symptoms of anemia appear. Even in drug-switching strategies, nausea/vomiting may also occur after switching, and these symptoms continue for a specific period unless the causative drug is discontinued. When nausea/vomiting were absent or when treatment was continued after the appearance of nausea/vomiting, treatment was continued until the end of the analysis period.

### Model Inputs

The parameters used in analyses are shown in Table 1.

**Table 1:** Model inputs

Item	Value	DSA		PSA			References
		Lower	Upper	Distribution	Parameters*		
Patient’s characteristic							
Age	40.7	–	–	–	–	–	[13]
*Transition probability*							
Incidence of nausea/vomiting							
SF 100 mg	31.0%	24.1%	37.9%	Beta	52.7	117.3	[13]
FC 500 mg	10.4%	7.2%	13.6%	Beta	35.88	309.12	[13]
FC 1000 mg	10.4%	7.2%	13.6%	Beta	35.88	309.12	[13]
Percentage of treatment discontinuation	47.0%	37.4%	56.6%	Beta	47.94	54.06	[11]
Utilities							
Without nausea/vomiting	0.945	0.756	1	Beta	4.555	0.265	[17]
Nausea/vomiting	− 0.117	− 0.1404	− 0.0936	Beta	88.183	665.518	[9]
Anemia	− 0.081	− 0.0972	− 0.0648	Beta	91.819	1041.749	[9]
Duration of nausea/vomiting symptoms (days/49 days)	9.7	6.9	12.5	Gamma	9.7	1.407	[13]
Drug costs (JPY/day)							
SF	10	–	–	–	–	–	Medical fee 2023
FC 500 mg	150	–	–	–	–	–	
FC 1000 mg	300	–	–	–	–	–	
*Productivity*							
Overall work impairment (for employees)							
With nausea/vomiting	53.5%	–	–	–	–	–	[10]
Without nausea/vomiting	32.2%	–	–	–	–	–	[10]
Daily activity impairment (for full-time housewives)							
With nausea/vomiting	54.3%	–	–	–	–	–	[10]
Without nausea/vomiting	36.6%	–	–	–	–	–	[10]
Average wages (JPY million / year)	416.85	-	-	-	-	-	[18]
Unpaid work of full-time housewives (JPY million / year)	460.2	-	-	-	-	-	[19]
Percentage of employees	79.4%	-	-	-	-	-	[20]
Percentage of full-time housewives	15.5%	-	-	-	-	-	[21]

*DSA* deterministic sensitivity analysis, *FC* ferric citrate hydrate, *PSA* probabilistic sensitivity analysis, *SF* sodium ferrous citrate

*Beta distribution (*α*, *β*), Gamma distribution (*α*, *λ*)

#### Transition probability

The incidence of nausea/vomiting with SF 100 mg/day was set at 31.0% based on the incidence of adverse drug reactions in the phase III study on FC [13]. The incidences of nausea/vomiting after administration of FC at 500 and 1000 mg/day were 12.1% and 7.6%, respectively. Since there was no dose-dependent increase in the incidence of nausea/vomiting with FC 500 mg/day or FC 1000 mg/day, the incidence of adverse drug reactions in the whole FC group (36 of 346 patients, 10.4%), and in the SF group (53 of 171, 31.0%) were used based on the incidence of nausea/vomiting [13]. The incidence of nausea/vomiting during treatment with each drug was assumed to be consistent regardless of whether the patients had previously taken any oral iron preparations. Therefore, the incidence of nausea/vomiting was set to be the same for both monotherapy and switching strategies (SF 100 mg/day, 31.0%; FC 500 mg/day and FC 1000 mg/day, 10.4%). The percentage of discontinuations due to nausea/vomiting caused by oral iron preparations was obtained from a web-based survey of general internal medicine and obstetrics/gynecology physicians [11]. In the survey, the 47% of patients discontinued treatment due to nausea/vomiting caused by oral iron preparations, based on responses from obstetricians and gynecologists.

#### Health utilities

The disutilities of nausea/vomiting and anemia symptoms were reported as − 0.117 and − 0.081, respectively. These values were derived from a web-based survey involving 385 patients who were taking iron preparations for HMB or anemia. The measurements were based on the 5-level EQ-5D version (EQ-5D-5L) [9]. The duration of nausea/vomiting was derived from the mean duration in the phase III study on FC [13]. In the study, the incidence of nausea/vomiting was significantly lower in the FC group than the SF group, whereas the duration of symptoms in cases with nausea/vomiting did not differ between the treatment groups (mean duration of 9.2 days among 36 cases in the SF group and, 10.0 days among 53 cases in the FC group). Therefore, the duration of symptoms for nausea/vomiting was set to be the same for SF 100 mg/day, FC 500 mg/day, and FC 1000 mg/day at 9.7 days per 7 weeks (9.2 × 36 + 10.0 × 53) / (36 + 53): the weighted mean duration of symptoms for nausea/vomiting cases in the SF group and FC group in the phase III study was used.

Health state utility without nausea/vomiting and anemia symptoms was set at 0.945 using the Japanese population norm for women in their 40 s [17].

#### Costs

Analyses from the Japanese public healthcare payer’s perspective only included drug costs of SF and FC. The cost of SF 100 mg, FC 500 mg, and FC 1000 mg were based on reimbursement prices as of April 2023 (JPY 10 per day, JPY 150 per day, and JPY 300 per day, respectively) [14]. Since treatment for nausea/vomiting was set to only be treatment discontinuation, additional costs due to nausea/vomiting were not considered. The cost of treatments for IDA other than oral iron preparations was not included, as they were assumed to be consistent across all strategies.

Analyses from limited societal perspectives included productivity losses during work for workers and losses from unpaid work in daily life for housewives in addition to drug costs for IDA. These societal costs were based on the findings of the Work Productivity and Activity Impairment survey of patients taking iron preparations for HMB or anemia [10]. This survey reported that overall work impairments in employment with and without nausea/vomiting were 53.5% and 32.2%, respectively, whereas daily activity impairments measured in all subjects were 54.3% and 36.3%, respectively [10].

### Analyses

The cost-effectiveness of FC treatment strategies was evaluated using the incremental cost-effectiveness ratio (ICER), calculated by dividing the incremental cost relative to SF 100 mg by the incremental QALYs relative to SF 100 mg. The threshold of ICER in the analyses was set at JPY 5 million/QALY [22]. Cases with ICER of JPY 5 million/QALY or less were rated as cost-effective.

To evaluate the uncertainty of each parameter used in the analyses, a deterministic sensitivity analysis (DSA) and probabilistic sensitivity analysis (PSA), which simultaneously change multiple parameters using a Monte Carlo simulation with 10,000 iterations, were conducted in the analyses from the Japanese public healthcare payer’s perspective. Sensitivity analyses were performed on comparisons of SF-FC 500 mg vs. SF 100 mg and FC 500 mg vs. SF 100 mg and all parameters, except for drug costs, were evaluated. The range of the confidence interval (CI) for DSA was 95%. If CI was not obtained, the range of the set value ± 20% of each variable was used as described in previous studies [23, 24]. PSA was assumed to have a beta distribution for transition probabilities and utility values, whereas a gamma distribution was applied for the duration of nausea/vomiting symptoms. Distributions were set using the standard error (SE) of each parameter. In some parameters, 10% of the set value was set as SE if it could not be estimated or was not available in the literature [23, 25].

## Results

The results of analyses are shown in Tables 2 and 3. Mean estimated QALYs were 0.4698 QALYs for SF-FC 500 mg and SF-FC 1000 mg, 0.4690 QALYs for FC 500 mg and FC 1000 mg, and 0.4646 QALYs for SF 100 mg. Incremental QALYs relative to SF 100 mg were 0.0052 QALYs for SF-FC 500 mg and SF-FC 1000 mg, and 0.0044 QALYs for FC 500 mg and FC 1000 mg. In the analyses from the Japanese public healthcare payer’s perspective, all strategies using FC had higher costs than SF 100 mg; incremental costs against SF 100 mg were JPY 5,775 for SF-FC 500 mg, JPY 11,769 for SF-FC 1000 mg, JPY 24,699 for FC 500 mg, and JPY 51,023 for FC 1000 mg. ICERs against SF 100 mg were JPY 1,107,780/QALY, JPY 2,257,477/QALY, JPY 5,588,430/QALY, and JPY 11,544,816/QALY, respectively (Table 2).

**Table 2:** Results of analyses from the public healthcare perspective

Strategies	QALYs	Δ QALY*	Costs (JPY)	Δ costs* (JPY)	ICER* (JPY per QALY)
SF 100 mg	0.4646	-	1,626	-	-
SF-FC 500 mg	0.4698	0.0052	7,401	5,775	1,107,780
SF-FC 1000 mg	0.4698	0.0052	13,395	11,769	2,257,477
FC 500 mg	0.4690	0.0044	26,325	24,699	5,588,430
FC 1000 mg	0.4690	0.0044	52,650	51,023	11,544,816

*FC* ferric citrate hydrate, *ICER* incremental cost-effectiveness ratio, *QALY* quality-adjusted life year, *SF* sodium ferrous citrate

*vs SF 100 mg

**Table 3:** Results of analyses from the limited societal perspective

Strategies	QALYs	Δ QALY*	Costs (JPY)	Δ costs* (JPY)	ICER* (JPY per QALY)
SF 100 mg	0.4646	–	747,579	–	–
SF-FC 500 mg	0.4698	0.0052	710,455	− 37,123	Dominant
SF-FC 1000 mg	0.4698	0.0052	716,449	− 31,130	Dominant
FC 500 mg	0.4690	0.0044	716,220	− 31,359	Dominant
FC 1000 mg	0.4690	0.0044	742,545	− 5,034	Dominant

*FC* ferric citrate hydrate, *ICER* incremental cost-effectiveness ratio, *QALY* quality-adjusted life year, *SF* sodium ferrous citrate. *Dominant* the QOLY is equal to or greater than that of the control and the cost is lower

*vs SF 100 mg

In consideration of productivity losses due to nausea/vomiting in the analyses from limited societal perspectives, all strategies using FC were cost-saving and superior to those using SF 100 mg. Saving costs for SF-FC 500 mg, SF-FC 1000 mg, FC 500 mg, and FC 1000 mg against SF 100 mg were JPY 37,123, JPY 31,130, JPY 31,359, and JPY 5,034, respectively (Table 3).

**Fig. 2:**
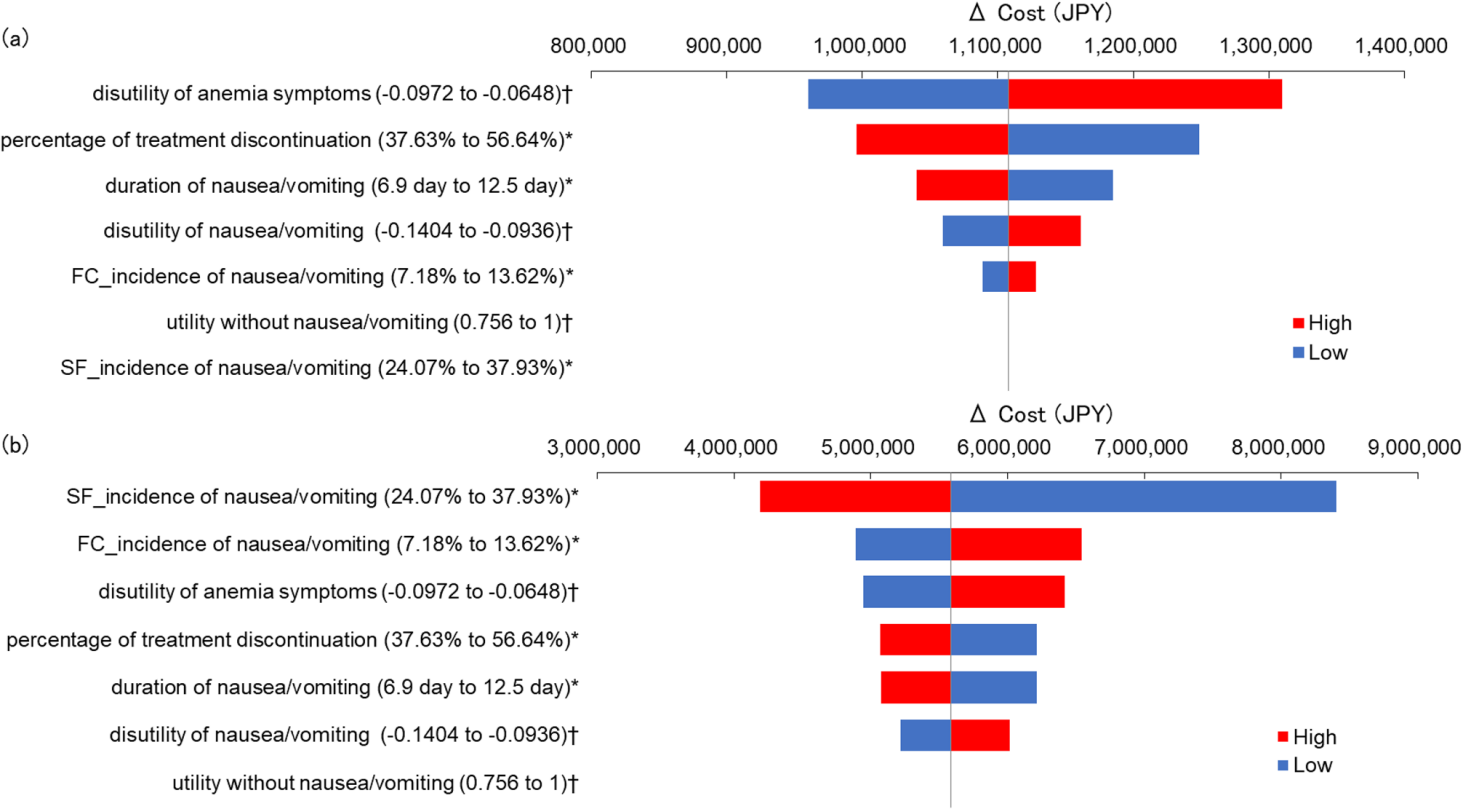
Results of deterministic sensitivity analysis. **a** SF 100 mg vs SF – FC 500 mg **b** SF 100 mg vs FC 500 mg. * 95%CI is used as the range of sensitivity analysis. † ± 20%CI is used as the range of sensitivity analysis. *CI* confidence interval, *FC* ferric citrate hydrate, *SF* sodium ferrous citrate

The results of DSA using a Tornado diagram are shown in Fig. 2. In the analysis of SF 100 mg vs. SF-FC 500 mg (Fig. 2a), the parameter with the greatest impact on ICER was “disutility of anemia symptoms”, although ICER was less than 5 million JPY/QALY for the range of both variables. In the analysis of SF 100 mg vs. 500 mg FC (Fig. 2b), “incidence of nausea/vomiting for SF 100 mg” was the parameter with the greatest impact on ICER. The ICER of FC 500 mg was below JPY 5 million/QALY when the incidence of nausea/vomiting for SF100 mg was ≥ 33.5%, the incidence of nausea/vomiting for FC500 mg was ≤ 7.7%, and disutility of anemia was ≤ − 0.096.

**Fig. 3:**
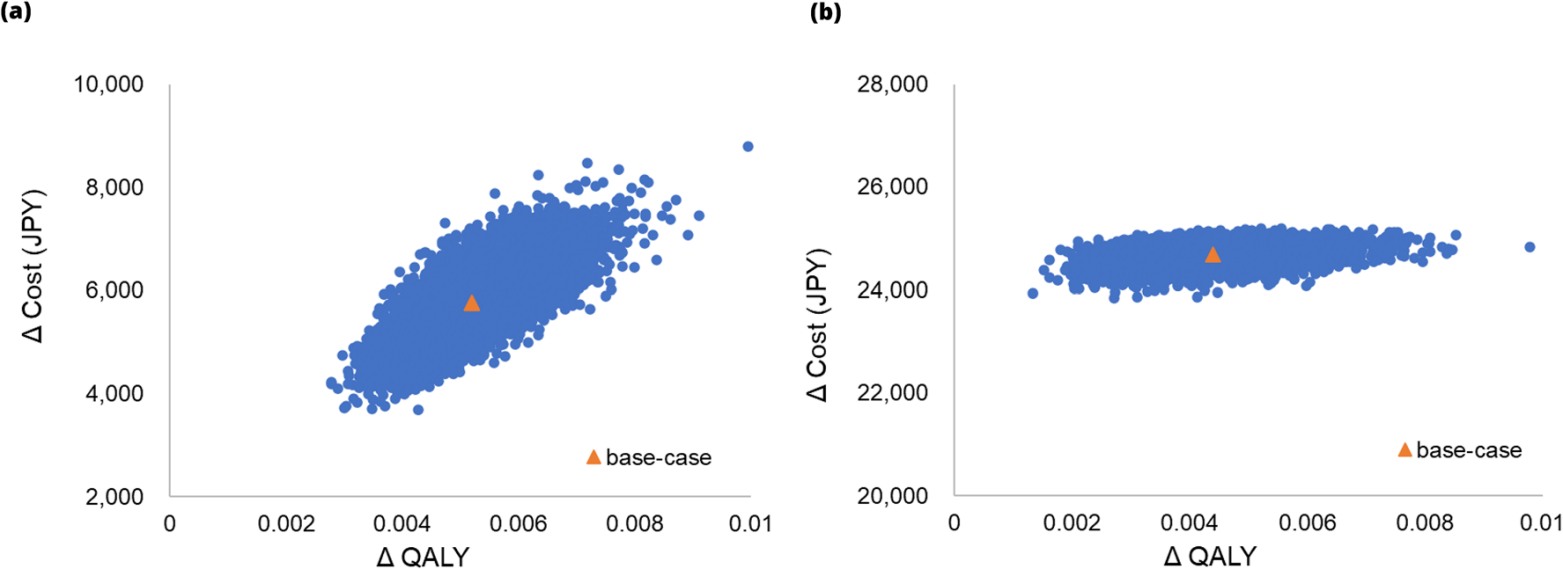
Probabilistic sensitivity analysis cost-effectiveness plane. **a** SF 100 mg vs SF – FC 500 mg **b** SF 100 mg vs FC 500 mg. The base-case plots the results of the base-case analysis, which **a** is JPY 1,107,780 per QALY **b** is JPY 5,588,430 per QALY. *FC* ferric citrate hydrate, *QALY* quality-adjusted life year, *SF* sodium ferrous citrate

**Fig. 4:**
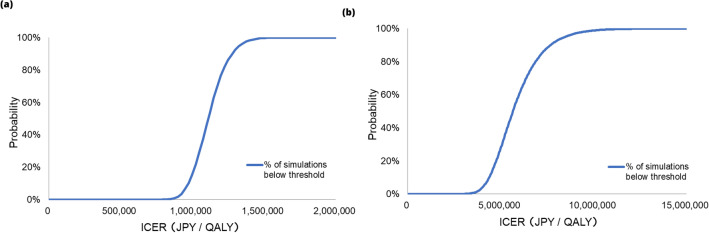
Cost-effectiveness acceptability curve. **a** SF 100 mg vs SF–FC 500 mg **b** SF 100 mg vs FC 500 mg. *FC* ferric citrate hydrate, *ICER* incremental cost-effectiveness ratio, *QALY* quality-adjusted life year, *SF* sodium ferrous citrate

The results of PSA showed that the probabilities of SF-FC 500 mg and FC 500 mg being more cost-effective than SF 100 mg were 100% and 27.8%, respectively, when the ICER threshold was set at JPY 5 million/QALY. Furthermore, the probabilities of FC 500 mg being more cost-effective was 88.4% and 98.8%, respectively, when the threshold of ICER was set at 7.5 million/QALY and 10 million/QALY. Scatter plots are shown in Fig. 3 and cost-effectiveness acceptability curves in Fig. 4.

## Discussion

In the present study, we compared the cost-effectiveness of four FC treatment strategies to that of the conventional treatment with SF at 100 mg and evaluated the health economical utility of FC. In all four FC treatment strategies, incremental QALYs were noted relative to SF at 100 mg. The ICER of the SF–FC 500 mg and SF–FC 1000 mg strategies, in which only patients who develop SF-induced nausea/vomiting were switched to FC 500 mg or FC 1000 mg, was lower than JPY 5 million/QALY in the analyses from the Japanese public healthcare payer’s perspective, which was favorable. In the analyses that considered productivity loss from limited societal perspectives, a cost reduction (dominant: the QOL is greater than that of the control and the cost is lower) was found in addition to an improvement in QOL in all four FC treatment strategies. In a comparison with a scenario in which treatment with SF was continued from health economical perspectives, these strategies were considered to be potent treatment options. On the other hand, strategies to initiate IDA treatment with FC at 500 or 1000 mg exceeded JPY 5 million/QALY in the analyses from the Japanese public healthcare payer’s perspective. Although the strategies improved patients’ QOL, patients used FC 500 mg or FC 1000 mg from the beginning regardless of the presence or absence of SF-induced nausea/vomiting, resulting in higher costs. However, cost reduction in addition to improvement in QOL (dominant) were found in the productivity loss-considered analyses from limited societal perspectives in those strategies.

Among the four FC treatment strategies, the drug-switching strategies from SF to FC (SF-FC 500 mg and SF-FC 1000 mg) showed the highest QALYs gained. This may have been because patients who developed SF-induced nausea/vomiting in the drug-switching strategies had the highest rate of patients in whom treatment for IDA could be continued in the 26-week analysis period. Since the discontinuation of treatment for IDA reduces QOL through the symptoms of anemia, QALYs gained may be maximized by inhibiting the rate of patients in whom treatment for IDA is discontinued. This result suggests the importance of continuous treatment for IDA to maintain QOL in patients with IDA.

Conventional oral iron preparations are generally regarded as adequate for the treatment of IDA; however, they may be associated with gastrointestinal adverse events, including nausea, vomiting, abdominal pain, and constipation, which can lead to treatment discontinuation [7, 11, 12]. Another option for IDA treatment is intravenous iron preparations; however, this treatment is highly invasive and adverse events, such as allergic reactions and hypersensitivity, may occur. In addition, time is required for hospital visits, which is not convenient for patients [26]. Therefore, there is a need for an oral iron preparation with fewer gastrointestinal adverse events, which may increase treatment adherence. FC, which has a lower incidence of gastrointestinal adverse events than conventional oral iron preparations, is expected to enable patients to continue treatment for IDA.

Nausea/vomiting related to iron preparations affect QOL and work productivity [9, 10]. Females aged 30–49 years account for the largest percentage of patients with IDA in Japan. Many women of this age are engaged in work and housekeeping/child rearing [27]. As this cost-effectiveness analyses included female IDA patients with a mean age of 40.7 years, the cost reductions could have been more clearly observed in all four FC treatment strategies that have a low risk of nausea/vomiting in the productivity loss-considered analyses from limited societal perspectives.

Within the four FC treatment strategies, QALYs gained and cost-effectiveness were favorable in the drug-switching strategies from SF to FC in the 26-week analysis period. However, when the analysis period was 14 weeks and analyses were conducted from limited societal perspectives, cost reductions in all four FC treatment strategies were maintained. With a shorter treatment period, incremental QALYs of treatment initiated with FC at 500 or 1000 mg, which have a low incidence of nausea/vomiting, were higher than those of the drug-switching strategies from SF to FC. When short-term treatment for IDA is needed or performed, first-line therapy with FC at 500 or 1000 mg may be a treatment option for patients requesting the earlier alleviation of effects on QOL or work productivity. In patients with IDA, treatment needs to be selected in accordance with better QOL, lower productivity loss and individual patient needs for treatment.

In these analyses, we considered work productivity loss with respect to the presence or absence of nausea/vomiting, but not the effects of anemia symptoms. In the present model, compared with FC, SF has higher proportion of patients discontinuing taking iron preparations and the anemia symptom is not ameliorated when discontinuing taking iron preparations, and therefore, work productivity loss due to lack of ameliorating of anemia symptoms in SF is assumed to be greater than in FC. Since productivity loss related to anemia symptoms was not considered in this study, cost reductions by treatment strategies initiating with FC may have been underestimated. IDA is reportedly associated with economic impairments because of physical productivity loss; therefore, the discontinuation of treatment for IDA may result in continuous anemia symptoms, leading to work productivity loss [28]. The four treatment strategies using FC may increase adherence by reducing the risk of oral iron preparation-induced gastrointestinal adverse events such as nausea/vomiting. As a result, the number of patients with attenuation of anemia symptoms may increase compared to that of patients with SF.

This study has a number of limitations that need to be addressed. First, although the patients gradually adapted, experiencing relief of symptoms of nausea/vomiting in the continuation of treatment, the present model assumes that nausea/vomiting continuously occurred for 9.7 days per 7 weeks until treatment switching or discontinuation. However, the results of the 14-week analyses were similar to those of the 26-week analysis; therefore, the effect on the results obtained herein may be weak. Second, there may be a specific number of patients in whom treatment with iron preparations did not improve the symptoms of anemia in actual clinical practice; however, we assumed that symptoms of anemia may be ameliorated with all the patients who continue the treatment with FC or SF in the present model’s structure. On the other hand, the proportion of the patients without improvements would be similar between FC and SF, if such patients did exist. Therefore, its effect on the present results was considered to be negligible. Third, the severity of nausea/vomiting and temporal changes in symptoms are not considered. In the phase III study [13], the severity of nausea/vomiting was evaluated, and all cases were mild or moderate, no severe case was observed. In contrast, the severity of nausea/vomiting was not considered in the present study, because the web-based survey data used to determine disutility could not obtain the values according to the severity of nausea/vomiting [9]. However, since there was no significant difference in the distribution of severity and duration of nausea/vomiting between the FC and SF groups in the phase III study [13], the temporal change in symptoms is assumed to be the same in both groups, the effect on the results obtained herein may be limited. In addition, the impact of disutility of nausea/vomiting was evaluated by deterministic sensitivity analyses (Fig. 2), and the results did not change over the range of the settings.

## Conclusion

Treatment with FC improved QOL for IDA patients. The cost-effectiveness of switching to FC was favorable from the public healthcare payer’s perspective, and switching to FC and monotherapy with FC contributed to cost reductions from limited societal perspectives. Moreover, the usefulness of FC was confirmed from the viewpoint of health economics. A therapeutic strategy for the treatment of IDA needs to be selected considering individual patient characteristics and cost-effectiveness.

## Data Availability

The datasets generated and/or analyzed during the study are available from the corresponding author on reasonable request.
